# Supplementary material for: Causal Relationship between Aging and Anorexia Nervosa: A White-Matter-Microstructure-Mediated Mendelian Randomization Analysis
Source: Biomedicines. 2024 Aug 16;12(8):1874. doi: 10.3390/biomedicines12081874 (PMC11351342; doi:10.3390/biomedicines12081874)
Supplement: Supplementary file 1 [file biomedicines-12-01874-s001.zip › Supplementary Figure/Figure S4/Singe SNP plot of MR analysis for TL to ubm-b-1471.pdf]

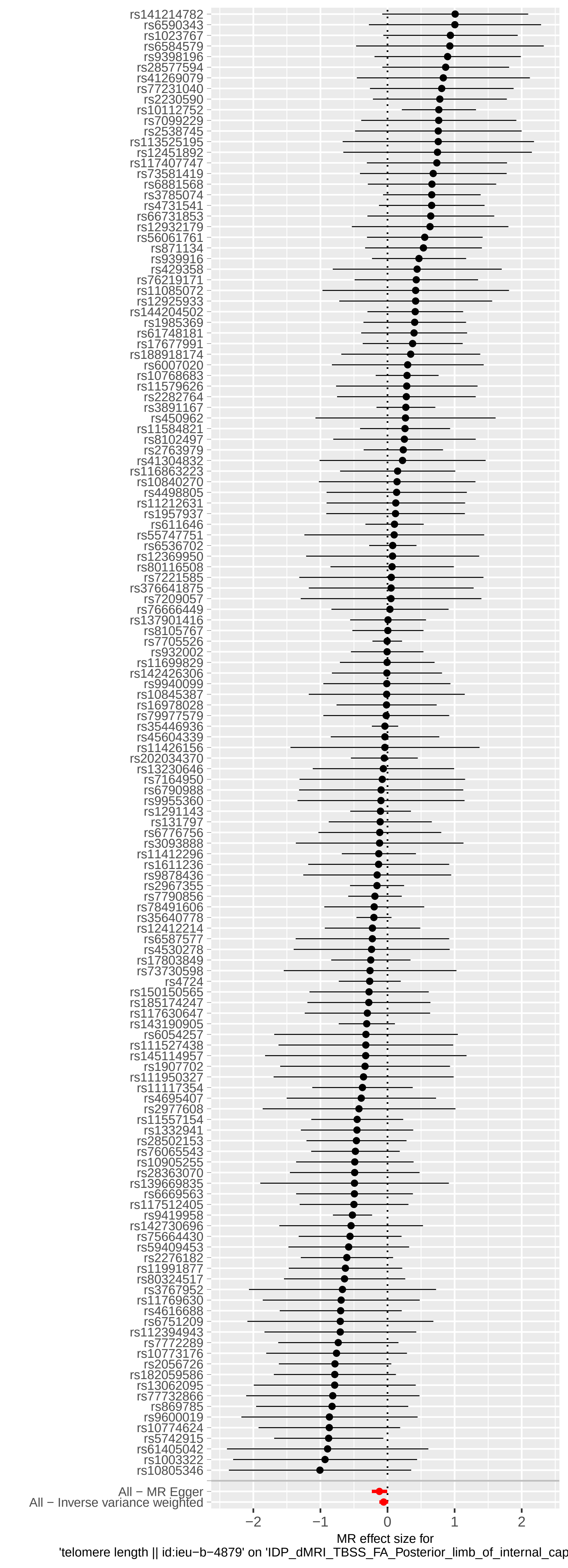
rs141214782
rs6590343
rs1023767
rs6584579
rs9398196
rs28577594
rs41269079
rs77231040
rs2230590
rs10112752
rs7099229
rs2538745
rs113525195
rs12451892
rs117407747
rs73581419
rs6881568
rs3785074
rs4731541
rs66731853
rs12932179
rs56061761
rs871134
rs939916
rs429358
rs76219171
rs11085072
rs12925933
rs144204502
rs1985369
rs61748181
rs17677991
rs188918174
rs6007020
rs10768683
rs11579626
rs2282764
rs3891167
rs450962
rs11584821
rs8102497
rs2763979
rs41304832
rs116863223
rs10840270
rs4498805
rs11212631
rs1957937
rs611646
rs55747751
rs6536702
rs12369950
rs80116508
rs7221585
rs376641875
rs7209057
rs76666449
rs137901416
rs8105767
rs7705526
rs932002
rs11699829
rs142426306
rs9940099
rs10845387
rs16978028
rs79977579
rs35446936
rs45604339
rs11426156
rs202034370
rs13230646
rs7164950
rs6790988
rs9955360
rs1291143
rs131797
rs6776756
rs3093888
rs11412296
rs1611236
rs9878436
rs2967355
rs7790856
rs78491606
rs35640778
rs12412214
rs6587577
rs4530278
rs17803849
rs73730598
rs4724
rs150150565
rs185174247
rs117630647
rs143190905
rs6054257
rs111527438
rs145114957
rs1907702
rs111950327
rs11117354
rs4695407
rs2977608
rs11557154
rs1332941
rs28502153
rs76065543
rs10905255
rs28363070
rs139669835
rs6669563
rs117512405
rs9419958
rs142730696
rs75664430
rs59409453
rs2276182
rs11991877
rs80324517
rs3767952
rs11769630
rs4616688
rs6751209
rs112394943
rs7772289
rs10773176
rs2056726
rs182059586
rs13062095
rs77732866
rs869785
rs9600019
rs10774624
rs5742915
rs61405042
rs1003322
rs10805346
All − MR Egger
All − Inverse variance weighted
−2
−1
0
1
2
MR effect size for
'telomere length || id:ieu−b−4879' on 'IDP_dMRI_TBSS_FA_Posterior_limb_of_internal_cap
